# IMRT using simultaneously integrated boost (SIB) in head and neck cancer patients

**DOI:** 10.1186/1748-717X-1-7

**Published:** 2006-03-31

**Authors:** G Studer, PU Huguenin, JB Davis, G Kunz, UM Lütolf, C Glanzmann

**Affiliations:** 1 Department of Radiation Oncology, University Hospital, Zurich, Switzerland; 2 Department of Radiation Physics, University Hospital, Zurich, Switzerland

## Abstract

**Background:**

Preliminary very encouraging clinical results of intensity modulated radiation therapy (IMRT) in Head Neck Cancer (HNC) are available from several large centers. Tumor control rates seem to be kept at least at the level of conventional three-dimensional radiation therapy; the benefit of normal tissue preservation with IMRT is proven for salivary function. There is still only limited experience with IMRT using simultaneously integrated boost (SIB-IMRT) in the head and neck region in terms of normal tissue response.

The aim of this work was (1) to establish tumor response in HNC patients treated with SIB-IMRT, and (2) to assess tissue tolerance following different SIB-IMRT schedules.

**Results:**

Between 1/2002 and 12/2004, 115 HNC patients have been curatively treated with IMRT. 70% received definitive IMRT (dIMRT), 30% were postoperatively irradiated. In 78% concomitant chemotherapy was given.

SIB radiation schedules with 5–6 × 2 Gy/week to 60–70 Gy, 5 × 2.2 Gy/week to 66–68.2 Gy (according to the RTOG protocol H-0022), or 5 × 2.11 Gy/week to 69.6 Gy were used.

After mean 18 months (10–44), 77% of patients were alive with no disease. Actuarial 2-year local, nodal, and distant disease free survival was 77%, 87%, and 78%, respectively. 10% were alive with disease, 10% died of disease. 20/21 locoregional failures occurred inside the high dose area. Mean tumor volume was significantly larger in locally failed (63 cc) vs controlled tumors (32 cc, p <0.01), and in definitive (43 cc) vs postoperative IMRT (25 cc, p <0.05); the locoregional failure rate was twofold higher in definitively irradiated patients.

Acute reactions were mild to moderate and limited to the boost area, the persisting grade 3/4 late toxicity rate was low with 6%. The two grade 4 reactions (dysphagia, laryngeal fibrosis) were observed following the SIB schedule with 2.2 Gy per session.

**Conclusion:**

SIB-IMRT in HNC using 2.0, 2.11 or 2.2 Gy per session is highly effective and safe with respect to tumor response and tolerance. SIB with 2.2 Gy is not recommended for large tumors involving laryngeal structures.

## Background

Preliminary very encouraging clinical results of IMRT in HNC are available from several large centers [1-6]. Tumor control rates seem to be kept at least at the level of conventional three-dimensional radiation therapy (3DCRT); the benefit of normal tissue preservation with IMRT is proven for salivary function; reduced dose exposure of the mandibular bone is described (manuscript submitted).

There is still only limited experience with simultaneously integrated boost (SIB) application in the head and neck region in terms of normal tissue response. As known from 3DCRT, dose, fractionation and treated volumes are the tumor control and normal tissue tolerance defining parameters. Dosimetric and volumetric relationships need to be newly defined for SIB, as the radiobiological response of intermediate dose volumes encompassing relatively small high-dose areas with increased doses per fraction seems to substantially differ from the situation in conventional techniques.

The intention of this prospective study was to present 3-year experiences in SIB-IMRT of HNC patients, focused on tumor response and tissue tolerance following different SIB schedules.

## Results

115 of 310 head and neck carcinoma (HNC) patients referred to our radiation oncology institution were treated curatively with IMRT (nasopharyngeal tumors excluded from analysis). The analysed patients were irradiated between January 2002 and December 2004; the mean follow up time was 18 months (10 – 44).

The median age was 60 years (15 – 85), with a male to female ratio of 3.4 : 1 (89 men, 26 women). The WHO Performance Status was 0 in 87, 1 in 26, and 2 in two patients. 71 patients (62 %) of the entire cohort presented with a T3/4 or T1-2/N2c, N3 tumor, 13 individuals (11 %) were referred for radiation of a recurrent tumor. Tumor subsites are listed in Table 1. The TN distribution consisted of 9 % T1, 28 % T2, 52 % T3/4 stages, and 11 % recurrent situations, respectively. 23 % of all patients presented with a N2c/3 nodal stage.

**Table 1:** Diagnoses and related primary tumor (T) stage distribution in 115 IMRT patients.

	Recurrence	T1	T2	T3	T4	Total
Oropharynx	3	7	16	16	14	56
Oral cavity	5	2	5	2	5	19
Hypopharynx	0	1	6	4	5	16
PNS	2	0	0	0	10	12
Supraglottic	0	0	5	1	1	7
Others	3	0	0	1	0	5
Total	13	10	32	24	36	115

PNS: paranasal sinus tumors others: thyroid (2), glottic (1), orbital (1) and parotid gland (1) tumors

The specific aims for performing IMRT were parotid gland sparing (n ~100), and/or mandible bone sparing (n = 76) and/or anterior visual pathway and/or brain sparing (n = 10).

34 patients (30 %, 30 following an R1 resection) were treated in a postoperative setting, 80 patients (70 %) underwent a primarily definitive radiation, re-irradiation after high dose 3DCRT was performed in one patient. One patient received preoperative irradiation.

Concomitant cisplatin based weekly chemotherapy (40 mg/m^2^, once a week, 1–7 cycles) was given to 89 patients (77 %). 61/89 patients (69 %) received 5 – 7 cycles (depending on the fractionation regime); 18 (20 %) underwent 4 cycles, 10 (11 %) only tolerated between 1 – 3 cycles. No treatment interruption was related to actinic toxicity; total treatment time was mean 46 days (33 – 60).

### Tumor response and survival

**Figure 1:**
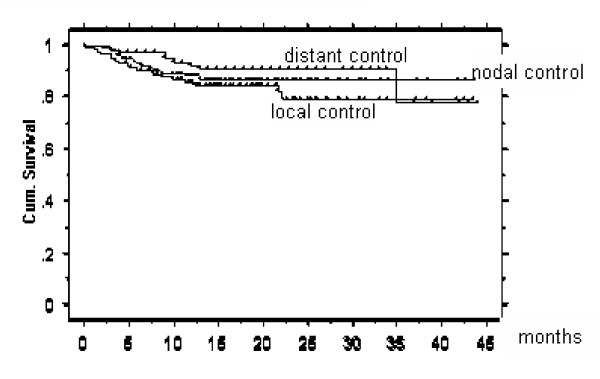
Actuarial 2 year local, nodal, and distant disease free survival: 77 %, 87 %, and 78 %, respectively

**Figure 2:**
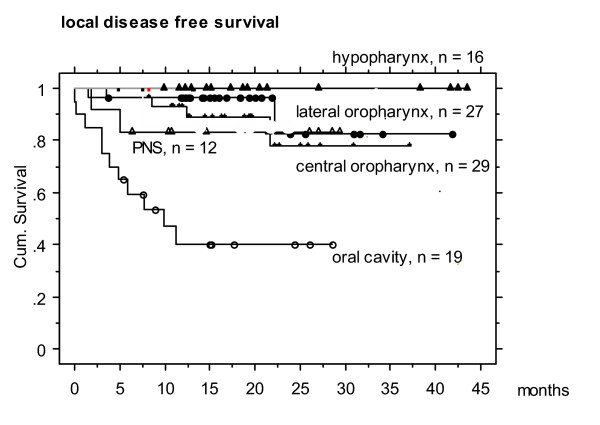
Actuarial 2 year local disease free survival in different HNC entities. Hypopharyngeal tumors revealed the highest local control rates, while oral cavity tumors showed the lowest rate. This fact can not be explained by TN stages or tumor volumes, and is issue of further data anaylses.

**Figure 3:**
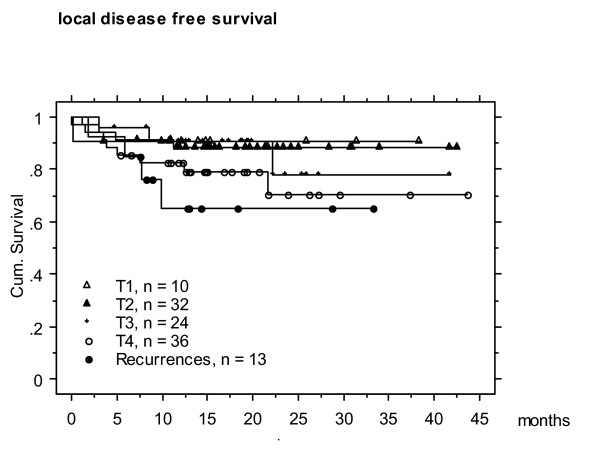
Actuarial 2 year local disease free survival according to the T-stages.

**Figure 4:**
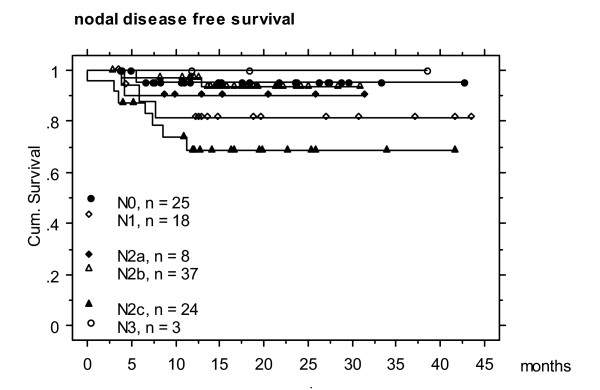
Actuarial 2 year nodal disease free survival according to N stages (N0 patients remain nodally controlled)

**Figure 5:**
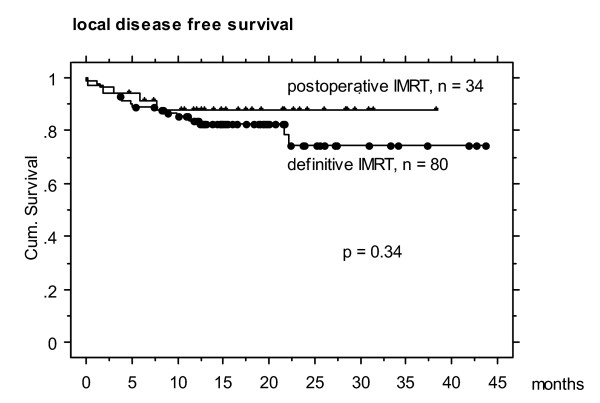
Actuarial 2 year local disease free survival in definitively vs postoperatively irradiated patients (non-significant difference).

Actuarial 2-year local, nodal and distant disease free survival was 77, 87 and 78 %, respectively (Figures 1- 5). At the time of data analysis (November 2005), 88/115 patients were alive with no evidence of disease (ANED, 77 %), 11 patients were alive with local and/or distant disease (AD, 10 %). 12/14 patients died of disease (DOD, 10 %), two died with intercurrent disease.

21/115 patients (18 %) experienced loco-regional failure (recurrence in 13, tumor persistence in 8, Table 2). 12/13 recurrences developed inside PTV1 ('in field', covered by > 95 % PTD), in one case marginal recurrence occurred in the distal, cervical aspect of the initial tumor arising from the floor of the mouth. No failure occurred related to/in the adjacent tissue of spared parotid gland.

**Table 2:** Characteristics on 21 patients (18 %) with loco-regional failure (LRF) are listed; patients with isolated distant failure (DF) are not included in this list. Mean time to failure (TTF) was 5 – 6 months in recurred patients; in 8 individuals (1/3) tumor persistence was observed.

Number	Diagnosis	TNM	LRF	DF	Outcome	TTF (m)	**GTV PT (cc)**	**GTV LN (cc)**	**PTV1 (cc)**	**%PTV <95**	**%PTV < 93%**
1	OC	T4N2c	LRR		AD	4	15	3	127	9	5
2	OC	T4N2c	LRR		DOD	10	75	27	253	6	3
3	OC	T1N2b	LR	distant	DOD	3	na	6.5	74	5	2
4	OC	T3N2c	LR		DOD	15	23	1	144	14	9
5	OC	T2N1	LR		AD	4	45	2	124	5	2
6	OC	Recurrence	LRR		AD	0	71	6	117	25	17
7	OC	T2N0	Persistence		AD	0	13	0	64	0	0
8	OC	T2N2c	Persistence		AD	0	16	2.4	82	50	7
9	OC	T4N1	Persistence		DOD	0	206	5	270	4	1
10	oro	T4N2b	LR	distant	AD	13	100	2	255	8	4
11	oro	T4N2c	NR	distant	DOD	3	34	15	179	8	4
12	oro	T4N0	Persistence		DOD	0	57	0	188	5	2
13	oro	T3N2b	Persistence	distant	AD	0	97	5	393	14	5
14	oro	T3N2a	Persistence		AD	0	31	4.3	198	35	25
15	oro	T4N2b	LRR		AD	8	41	5	178	15	10
16	Sinus	T4N0	Persistence		DOD	10	75	0	75	8	5
17	Sinus	Recurrence	Persistence	distant	AD	0	56	20	89	27	11
18	Sinus	T4N2b	LRR		DOD	15	141	17	176	11	5
19	Glottic	Recurrence	NR	distant	DOD	13	9	118	8	3	3
20	Supragl	T4N2c	LRR	distant	AD	6	79	18	353	7	2
21	Hypoph	T3N2c	NR		ANED *	9	22	30	210	15	7
**Mean**						**5.4**	**63.0**	**8.5**	**174.6**	**13.3**	**6.1**
**Range**						**0 – 21**	**9 – 206**	**0 – 99**	**64 – 353**	**0 – 50**	**0 – 25**

LRF loco-regional failure; DF distant failure; LC local recurrence; LRR loco-regional recurrence; NR nodal recurrence; TTF time to failure; GTVPT primary gross tumor volume, GTV LN lymph node gross tumor volume; PTV1 planning target volume 1 (boost).

In loco-regionally failed cases, doses < 95 % were delivered to mean 13.5 % (0 – 50) of PTV1, vs mean ~8 % (0 – 24) in loco-regionally controlled individuals (p > 0.5, Table 3). 5 loco-regionally controlled patients suffered from distant failure.

**Table 3:** Volumetric characteristics of loco-regionally failed (LRF) vs loco-regionally controlled (LRC) patients without vs with late term reactions grade 3/4.

	LRF	LRC, G 0–2	LRC, G 3–4
n	21	77	14*
GTV PT (cc)	63	32	31.4
GTV LN (cc)	8.5	15	13
PTV1 (cc)	174	154	176
% PTV1 >110 %	0.8	0.8	1.3
% PTV1 < 95 %	13.5	8.3	8.0

Gross tumor volume (GTV) in LRF patients was significantly larger than in controlled LRC individuals (p < 0.01). Isodose comparison showed PTV1 in controlled patients tendentially better covered, with less volumes getting doses < 95 %, compared with failed patients.

* : the 2 patients with xerostomia grade 3 and the 3 patients with ulcers related to tumor persistence were excluded from this analysis.

Local failure occurred twice as often in definitively as in postoperatively irradiated patients, with 15/80 (19 %) vs 3/34 (9 %) (Figure 5), respectively; nodal failure rate was 11/80 (14 %), vs 1/34 (3 %) distant failure rate 6/80 (8 %) vs 4/34 (12 %), respectively. Tumor volumes in the definitive vs postoperative IMRT subgroup differed significantly with mean/median 43/32 cc (3 – 205) vs 24.7/14 cc (2 – 74), respectively (p < 0.05).

The primary GTV measured mean 38.2 cc (2 – 206), the nodal GTV mean 12 cc (1 – 70). The mean volume of the primary GTV in patients who failed locally was 63 cc (13 – 206) and differed significantly from mean 32 cc (range 2 – 124) in locally controlled patients (p < 0.01, Table 3).

### Early toxicity

Xerostomia grade 3 was observed in 10 % of patients at completion of treatment. Mucositis (15 % grade 3), and dermatitis (5 % grade 3) were limited to the high dose volume. Grade 3 dysphagia developed in only 20 % of the cases, translating into an improved patient's performance status during treatment (QoL analysis in preparation). No grade 4 early reaction, and no radiation-toxicity related treatment interruption occurred.

A gastric feeding tube was used in 37 patients (33 %), in the majority of them prior to IMRT start because of pre-treatment weight loss due to pain or tumor-related mechanic dysphagia. The mean weight loss at completion of IMRT was 6 % (range: 25 % loss to 15 % gain under treatment); 19/113 patients (17 %) lost ≥ 10 % of their initial weight; one third of them despite feeding tube (>10 % loss in 20 % of patients of whom feeding tube was inserted in 33 %). 42 % of all patients kept pre-treatment weight (n = 45) or gained weight under treatment (n = 8).

### Subacute and late toxicity (> 90 days from treatment completion)

19 (18 %) grade 3/4 subacute or late effects (included 2 cases with a grade 3 xerostomia) in 18 out of 109 individuals treated with SIB-IMRT, were observed so far (Table 4); all lesions were localized in the high dose SIB area (PTV1, mean 176 cc, range 78 – 299), and developed 2 – 12 months after SIB-IMRT completion. This includes a dysphagia grade 4, a laryngeal fibrosis grade 4 requiring a permanent tracheostoma, an osteo-radionecrosis grade 3 of the mandible, which was resolved by lingual bone decortication, grade 3 dysphagia in 2 cases, grade 3 xerostomia 1 year after IMRT in 2 (in one of them no parotid gland sparing was performed), and mucosal ulcers in 12 cases.

**Table 4:** Characteristics on patients with grade 3/4 late term effects (19 events in 18 patients). In all cases with grade 3/4 ulcers not healing during a 6 months period (n = 3, grey bars), ulcer persistence was found basing on tumor persistence (No 3,13, 16; data from these patient as well as of the 2 individuals with grade 3 xerostomia were excluded from this volumetric analysis (EA) of the 14 patients with grade 3/4 lesions).

								Outcome							
No.	Dg	TNM	Sequence	Grade 3/4	t post RT (m)	Duration (m)	Treatment	NTR	Tumor	PTD	d/f SIB	Dmax G3/4	GTV PT (cc)	PTV1 (cc)	cc>110% D

1	Cent oro	T3N2c	Prim	Ulcer	4	7	-	Healed	ANED	66/54	2.2	75.7	56	213	0
2	Supragl	T2N2b	Prim	Ulcer	6	1	-	Healed	ANED	69.6/54	2.11	75.9	20.8	162.5	0
3	Oral cav	T2N0	Postop	Ulcer	3	4	0	Healed	ANED	66/54	2	80.7	14	81.7	1.6
4	Hypo	T4N1	Prim	Ulcer	3	3	HBO	Healed	ANED	66/54	2.2	75.8	74	299	0
5	"	"	"	Larynx fibrosis	10	Persistent (30)	Tracheostoma	Tracheostoma	ANED	"	2.2	75.8	"	"	0
6	Hypo	T2N2b	Prim	Ulcer	4	5	-	Healed	ANED	69.6/54	2.11	74.8	27	145	0
7	Cent oro	T3N2b	Prim	Ulcer	6	1	-	Healed	ANED	66/54	2.2	77.3	30	201	2
8	Hypo	T2N2b	Prim	Ulcer	6	2	-	Healed	ANED	69.6/54	2.11	76.8	34.5	220	2.2
9	Cent oro	T3N0	Prim	Ulcer	4	3	HBO	Healed	ANED	66/54	2.2	77.3	29	77.7	7.7
10	Lat oro	T3N2b	Postop	Ulcer	2	3	-	Healed	ANED	65.4/54	2.11	72.8	7.8	212	0
11	Cent oro	T3N2b	Prim	Bone	4	6	Surgery	Healed	ANED	66/54	2.2	76.5	37.5	208	2
12	Hypo	T3N0	Prim	Dysphagia	2	? (8)	Dilatation	Persistent	ANED	69.6/54	2.11	75.7	32	149.5	0
13	Hypo	T3N2c	Prim	Dysphagia	5	Persistent (14)	Dilatation	Persistent	ANED	68.2/54	2.2	79.2	21.5	210	2.1
14	Cent oro	T3N2b	Prim	Dysphagia	5	? (9, lost)	-	?	ANED	69.6/54	2.11	76.7	24	113	0
**15**	**Oral cav**	**T2N2c**	**Prim**	**Ulcer**	**3**	**Persistent**	**-**	**Persistent**	**TU Persistent**	**69.6/54**	**2.11**	**EA**	**EA**	**EA**	**EA**
**16**	**Lat oro**	**T2N1**	**Prim**	**Ulcer**	**2**	**Persistent**	**-**	**Persistent**	**TU Persistent**	**69.6/54**	**2.11**	**EA**	**EA**	**EA**	**EA**
**17**	**Cent oro**	**T3N2b**	**Prim**	**Bleeding ulcer**	**0**	**Persistent**	**Surgery**	**Persistent**	**TU Persistent**	**69.6/54**	**2.11**	**EA**	**EA**	**EA**	**EA**
18	Hypo	T4N2a	Prim	Xerostomia	0	Persistent (14)	-	Persistent	ANED	69.6/54	2.11	EA	EA	EA	EA
19	Cent oro	T1N2b	postop	Xerostomia	0	Persistent (12)	-	Persistent	ANED	64/54	2	EA	EA	EA	EA
Mean					3.6	3.2						76.5	31.4	176.3	1.3
Range					0–10	1–7					2.0–2.20	72.8–80.7	2.5–37.5	78–299	0–7.7

t postRT time (in months) from IMRT completion to appearance of late term reaction

NTR normal tissue reaction

PTD prescribed total dose

The most frequent grade 3/4 late term effect was mucosal ulceration in the area of the SIB. This was characterized by its appearance mean 4 months (2 – 6) after IMRT completion, by its persistence for mean 3 months (1 – 7), and spontaneous healing in all locally controlled cases. All ulcers occurred in oro-hypopharyngeal and oral cavity tumor patients, no ulcer was observed in paranasal sinus or nasopharyngeal cancer patients. In 3 patients who suffered from persisting ulceration for a period longer than 7 months, underlying tumor persistence was histologically confirmed 8, 10 and 11 months after completion of treatment. One of these three patients experienced substantial ulcer bleeding from the large tumor ulceration which was already present before IMRT start.

In grade 3/4 event patients (Table 4), mean 1.3 % (0 -10 %, or 0 – 7.7 cc) of the entire PTV1 received more than 110 % of the prescribed total dose. In 9 of the 19 cases, maximal doses were below 110 %; in only 4/19 patients, a hot spot area was matching with the area of a grade 3/4 tissue lesion.

The patient with grade 4 laryngeal fibrosis became symptomatic after a latency of 12 months following treatment with SIB ^2.2 ^to 66 Gy for a large T4 hypopharyngeal cancer that involved the oropharynx, hypopharynx and larynx. No hot spot was delivered to the area of the actinic lesion. 3.5 years post treatment, this patient is free of disease.

The 3 patients with grade 3/4 dysphagia were treated for extended T3 primaries of the hypopharynx (2) and oropharynx (1); all three affected patients are women. After follow up periods of 9 and 14 months, no improvement was observed in two; a third patient was lost of follow up 9 months after treatment completion.

SIB-IMRT resulted in a 1-year swallowing / salivary function of grade 0 -1 dysphagia / xerostomia in 95 / 80 % (n = 77). In only 2 patients, less than 30 % of the total parotid gland volume (both parotid glands = 100 % volume) could be kept below mean doses of 26 Gy; in 74 % of the patients the spared glandular total volume ranged between 60 % and 100 %, in ~25 % of the patients, the protected glandular volume ranged between 30 and 60 % (Figure 7 and Figure 8 illustrate an example of spared total parotid gland volume of 62 %).

When late reactions are analysed according to the different SIB schedules, the following distribution was found: 7 events developed in the 33 SIB ^2.2 ^cases (21 %), 10 events in the 47 SIB ^2.11 ^(21 %), and 2 in the 22 of 29 SIB ^2.0 ^patients (9 %) with doses > 65 Gy.

In locally controlled patients, 6 persistent late effects were observed: xerostomia (2), laryngeal fibrosis (1), and dysphagia (3), last assessed at 14 months, 3.5 years, and 9 – 17 months after completion of IMRT, respectively. This translates into a grade 3/4 toxicity rate of ~6 % (5/80) in the SIB^2.11/2.2 ^subgroup, or of 5.5 % (6/109) in the entire SIB-IMRT cohort, respectively.

At one year post treatment, mean weight loss was 4 % (range minus 24 % to plus 13 % of pre-treatment value); 7/77 patients with 1 year follow up still had ≥ 10 % less weight than before treatment, 18 patients reached their initial weight or more (n = 10).

## Discussion

### Disease control

The high 2-year locoregional disease free survival as well as the locoregional failuare pattern in our patients is comparable to the excellent results reported in the literature on IMRT of head and neck tumors (Table 5). Most of these results are superior to historic results following 3DCRT series with disease free survival rates ranging between about 40 and 88 % [4,7].

**Table 5:** Disease outcome following IMRT in selected published series including the own study

Authors	HNC cohorts	N patients	LC (%)	NC (%)	LRC	DC (%)	OAS (%)	time point
Eisbruch et al [11]	oro/hypo/OC	133			94/77/60			3y
Dawson et al [1]	HNC w/o NPC	58			79			2y
Own study	HNC w/o NPC	115	77	87		78	86	2y
Eisbruch et al [11]	dIMRT/pIMRT	60/73			81/84			3y
Chao et al [4]	dIMRT/pIMRT	31/43			78/95	84/94	87	3y
Chao et al [5]	dIMRT/pIMRT	52/74			79/90			2y
own study	dIMRT/pIMRT	80/34	81/91	86/97		92/88	75/79	2y
Eisbruch et al [11]	oro	80			94			3y
Garden et al [in 6]	oro	80 (T1-2N0)			94			2y
De Arruda 17	oro	50	98	88		84	98	2y
Huang et al [in 6]	oro	41	94		89	91	89	2y
Own study	oro	56	88	93		93	87	2y

LC local control; NC nodal control; LRC loco-regional control; DC distant control, OAS overall survival; oro oropharyngeal tumor; OC oral cavity tumor; NPC nasopharyngeal cancer; d/pIMRT defintive/postoperative IMRT.

Operated patients in our cohort showed half as large tumors and half the local recurrence rate as primarily irradiated patients. The significant correlation between tumor size and tumor control is shown by several investigators [8,9].

Dawson et al reported on 12/58 failed patients (21 %), of whom 10 /12 relapsed in-field, two marginally [1]. Of 17/126 (13 %) failures in Chao's et al's series [5], 9 were inside the CTV1, one was marginal, one outside the CTV1 but inside CTV2.

Considering own and published results on locoregional failure analyses [1,5,10,11], one can conclude that the volumetric concept used so far in HNC IMRT is appropriate, and the loco-regional control can hardly be improved by volumetric optimisation.

### Acute tolerance

Grade 3 mucositis, dermatitis, and dysphagia rates were 15 %, 5 %, and 20 %, respectively, comparing with 50 % to more than 80 % acute mucositis [12-15], and ~33 % up to 50 – 70 % dysphagia [7,15,16] in 3DCRT.

De Arruda et al reported 38 % grade 3 mucositis in 50 SIB-IMRT patients, and 6 % grade 3 skin reactions; 62 % developed grade 3 acute reactions [17]. Chao et al [4] found 37 % grade 3/4 skin toxicity, 40 % grade 3/4 mucositis in 74 oropharyngeal cancer patients necessitating a gastrostomy tube during chemo-IMRT in 23 %.

Mucosal and dermal acute reactions occurred only localized and healed up faster in our IMRT patients than used in 3DCRT patients. Only few patients presented with an acute grade 3 mucositis in the boost area. This phenomenon is not entirely understood and may be related to improved tissue tolerance when only moderate doses are delivered to adjacent tissue areas.

### Late tolerance

12 subacute grade 3/4 mucosal ulcers in the PTV1 were observed, which were characterized by self-limitation and spontaneous healing. 8/19 patients with late reactions were exposed to > 110 % of prescribed total doses, in only 4 of them hot spots matched with the area of the actinic lesion, indicating the hot spots not to be the main reason for these lesions.

Xerostomia grade 3 at 1 year was scored in 2 (3 %) patients at risk; 3 patients at risk developed dysphagia grade 3/4. In a group of 50 patients, De Arruda et al observed 8 cases (16 %) of pharyngeal grade 3 reactions in the MSKCC IMRT series; three patients developed cervical esophageal stricture requiring dilatations [17]. In a 3DCRT study by Huguenin et al [7], higher incidences of 12 % and 22 % were reported for xerostomia and dysphagia, respectively. Dysphagia/aspiration related structures have been investigated by Eisbruch et al [18]. Pharyngeal constrictors, glottis and supraglottic larynx have been identified as the anatomic correlates whose damage may cause the symptoms. IMRT can moderately spare these structures; if substantially affected by tumor, hot spots and probably also SIB doses > of 2.0 Gy per fraction should be avoided. Consequently, we avoid SIB^2.2/2.11 ^in patients where the tumor affects major parts of the larynx.

In ~75 patients at risk, one grade 3 osteonecrosis, treated without mandible resection, was diagnosed 4 months after IMRT completion. In 3DCRT, the incidence of osteo-radionecrosis is higher by approximately 4–6 % after 2 years [19], although FU of the presented IMRT cohort is still short for definitive result.

### SIB-IMRT

The advantage of SIB-IMRT consists in a better target conformity [20-24], less dose to critical structures, moderate treatment acceleration with reduced total treatment time, and the option of dose escalation in the gross tumor volume.

There is limited experience in normal tissue tolerance following SIB-IMRT in HNC.

Many different SIB schedules (references [2,17,22-29], two RTOG protocols (H-0022 and 0225)) have been published; to this date there is no universally agreed standard of dosage.

We found SIB ^2.11 ^and SIB^2.2 ^equally well tolerated and safe with respect to acute and late normal tissue tolerance compared to 3DCRT, except of the described grade 4 reactions when 2.2 Gy per session delivered to larger laryngeal areas. The weakness of this comparison lies in its retrospective approach.

The unexpected observation of very few (~15 %) cases with grade 3 acute mucositis despite full SIB dose delivered to the mucosa, and observed better tissue healing, are interesting and clinically relevant findings that may indicate a higher tolerance, when surrounding tissue volumes are exposed to lower doses. This phenomenon has been described decades ago, based on the clinical observation of the so called 'grid therapy' [30-34], a technique used to deliver high single fraction doses of radiation by converting a large treatment field into many smaller fields. The use of this technique goes back to the beginning of the last century when orthovoltage radiation was mainly used for external beam radiation therapy. Small areas of skin within an irradiated field, shielded from direct radiation, are reported to serve as centers for re-growth of normal skin tissue, and allowed up to six times the conventional open doses without an increase in skin reactions or complications to underlying structures.

Moreover, grade 3/4 late effects could not be related to hot spots in the majority of our cases, indicating additional factors determining normal tissue tolerance in IMRT.

With respect to future proceeding, mild dose escalation limited to the GTV in patients with intermediate tumor volumes and related intermediate disease outcome, respectively (manuscript submitted: disease outcome related to GTV), is in evaluation as a first consequence of these data.

## Conclusion

IMRT in HNC, using the planning target volume and dose concept as described, is a highly effective technique with respect to tumor response and tolerance. SIB-IMRT is safe and similarly well tolerated using either 2.11 or 2.2 Gy per fraction to total doses of 66–70 Gy, although is not recommended for large tumors involving laryngeal structures.

There is clinical evidence for increased normal tissue tolerance following IMRT.

## Methods

### SIB schedules

SIB was performed in 109/115 patients; in the remaining six cases a single dose-volume was painted.

### Biomathematical consideration

In order to employ a slightly accelerated SIB schedule, 30 × 2.2 Gy per fraction, 5× per week, to 66 Gy in the high dose area (PTV1), was chosen. This corresponds with the BED of 35 × 2 Gyper session, 5x / week, to 70 Gy in terms of early and late tolerance, assuming an alpha value of 0.35, and an alpha/beta ratio of 10 and 3, respectively (BED for late effects 116.66, BED for early effects 70.1 Gy). Similarly, 2.11 Gy per fraction in 33 sessions to 69.6 Gy (PTV1) equals with 35 × 2 Gy to 70 Gy.

SIB-IMRT technique was performed using the following schedules (5 fractions/week each):

2.2 Gy (PTV1) / 1.8 Gy (PTV2) to 66 Gy / 54 Gy, 5 fractions/week (n = 33, SIB^2.2^)

2.11 Gy (PTV1)/1.64 Gy (PTV2) to 69.6 Gy / 54 Gy, 5 fractions/week (n = 44, SIB^2.11^)

2.11 (PTV1) / 1.8 Gy (PTV2) to 63.3 / 54 Gy, 5 fractions/week (n = 3, SIB^2.11^)

2.0 Gy (PTV1)/ 1.5–1.8 Gy (PTV2) to 60 – 70 / 52–56 Gy, 5–6 fractions/week (n = 34, SIB^2.0^)

In one patient with large necrotic nodes, a higher SIB dose of 2.35 Gy per fraction to 75.2 Gy was delivered.

During the first 20 months, SIB-IMRT was performed with SIB^2.2 ^according to the RTOG study protocol H-0022.

Intermediate doses were individually defined to regions considered at high risk for microscopic disease (PTV3, doses ranging from 56 – 60 Gy).

In 7 / 33 patients subacute mucosal ulcers were observed. As a consequence the decision was made to change the SIB^2.2 ^schedule to a slightly less accelerated schedule with 2.11 / 1.64 Gy per fraction to 63.3 – 69.6 / 54 Gy in 30 – 33 fractions (n = 47).

In all patients with tumor extension close to, or invading the central nervous system (CNS), and in most patients treated in a postoperative setting (n = 22/34), SIB^2.0 ^was prescribed. Doses to CNS structures never exceeded 2.0 Gy per fraction and 70 Gy total dose, respectively.

### Planning Computerized Tomography (Planning CT)

Planning CT (Somatom Plus 4, Siemens) was acquired with 2 – 3 mm slice thickness and no interslice gap throughout the whole sequentially acquired region of interest. Patients were immobilized in a commercially available thermoplastic mask with fixed head and shoulder. An integrated individually customized bite block.

In patients with postoperative irradiation gross tumor volumes were drawn slice by slice in the planning CT, based on diagnostic preoperative MRIs and PET-CTs, which were available for all patients. In the majority of the definitively irradiated patients, fused 'PET-Planning CTs' were performed.

### Planning systems

Contouring and plan optimisation was performed on a Varian Treatment Planning System (Eclipse^®^, Version 7.3.10, Varian Medical Systems, Hansen Way, Palo Alto CA, 94304-1129)

### Delineation of planning target volumes (PTVs)

#### Definitions

Gross Tumor Volume (GTV) with a margin of 10–15 mm was included in the SIB volume (PTV1, 60 – 73 Gy)

Elective lymph node regions (PTV2, doses between 48 – 56 Gy):

In hypopharyngeal, central oropharyngeal and lateral oropharyngeal tumors extending to midline structures, bilateral lymph node regions level 2 – 5 and retropharyngeal nodes were included.

In lateral oropharynx tumors with bilateral nodal disease, bilateral nodes level 2 – 5 were irradiated. In cases with minimal contralateral nodal disease, level 2 – 5 excluding the uppermost part of contralateral level 2 was included. In nodally negative lateral T1-2 oropharynx tumors without infiltration of the tongue and without palatinal infiltration crossing the midline, the elective node irradiation was limited to the ipsilateral side. In T3/4 N0 or ispilateral N1/2 situations, ipsilateral level 2 – 5 and contralateral level 2 – 4 without the uppermost part were included, respectively.

Submandibular nodes have been electively included only in oral cavity tumors, or in tumors extending to the oral cavity.

**Figure 6:**
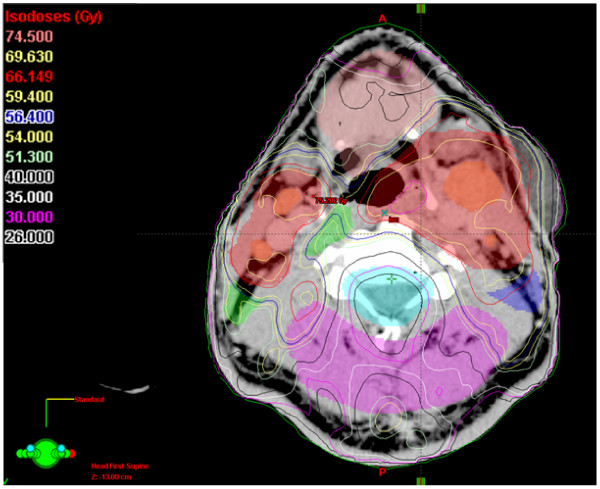
An example of an IMRT isodose plan using simultaneously integrated boost. Depicted is an axial slice, 64 mm above the isocenter of the plan. Contoured are PTV1 (69.6 Gy), PTV2 (60 Gy) and PTV3 (54 Gy), gross tumor volumes of the primary and macroscopic nodal disease, and normal structures (spinal cord, brain, parotid glands, anterior soft tissues, dorsal soft tissues). Note the well-spared spinal cord and parotid glands despite of bilateral nodal disease covered with high doses (nodal and primary gross tumor volumes included into the PTV1).

**Figure 7:**
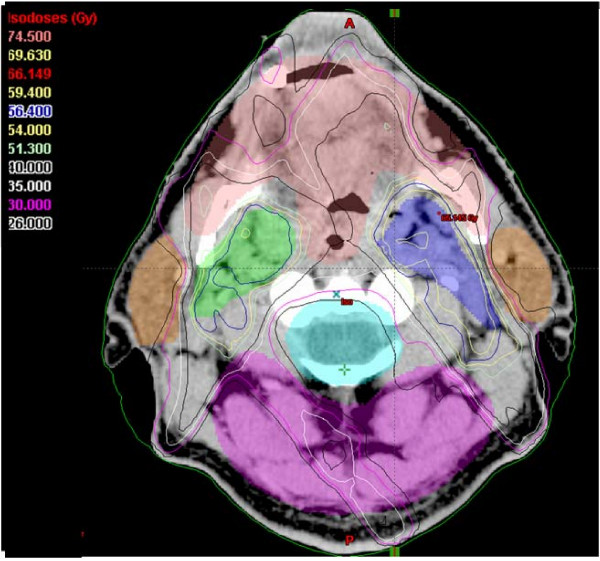
An example of an IMRT isodose plan using simultaneously integrated boost. A more distal axial slice 12mm above the isocenter

**Figure 8:**
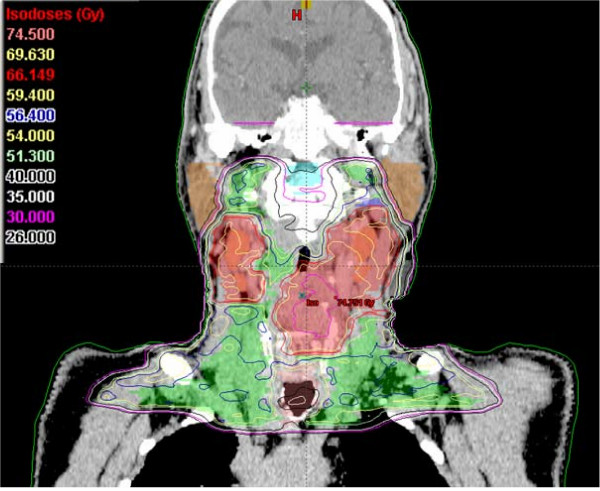
An example of an IMRT isodose plan using simultaneously integrated boost. A sagital view of a T2N2c staged hypopharyngeal cancer patient.

**Figure 9:**
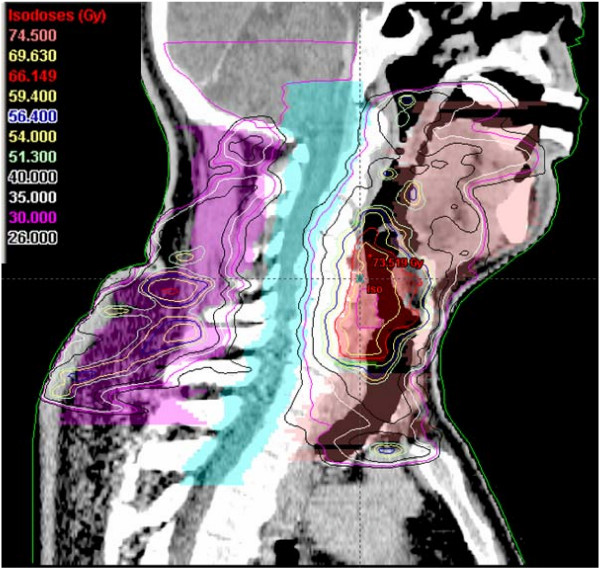
An example of an IMRT isodose plan using simultaneously integrated boost. A coronar view of a T2N2c staged hypopharyngeal cancer patient.

Dose constraints for normal tissues / organs at risk (OARs) outside PTVs (see also Figures 6, 7, 8, 9)

Dose planning aimed at target doses of 60 – 70 Gy. Normal tissue doses were defined as follows:

Spinal cord/brain stem: maximum dose (Dmax) < 45 Gy, mean dose (Dmean) < 35 Gy (spinal cord was contoured with an at least 5 – 10 mm margin, > 10 mm at the ventral aspect)

Parotid (entire or partial) gland volume, spared to the degree possible without compromising target coverage: Dmean < 26 Gy (outlined was the partial volume provided to be spared, no overlapping with PTVs; contouring of the entire glands for analytic purposes)

Optic nerve outside PTV: Dmax < 50 Gy (optic nerve, retina and chiasm were contoured with a safety margin of 2 – 4 mm)

Chiasm: Dmax < 50 Gy

Retina outside PTV: Dmax < 45 Gy

Lacrimal glands: Dmax < 30 Gy

Brain: depending CNS vicinity to the tumor; Dmax ≤ 100 % of prescribed total dose of maximal 70 Gy, doses per fraction of 1.8–2.0 Gy

Temporomandibular joint (TMJ): Dmax < 50 Gy

Oral cavity outside the PTV (contouring included the mandible and maxillary bone and the oral vestibulum): Dmean < 35 Gy

Nuchal tissue: Dmean < 45 Gy

### Radiation

Irradiation was delivered by 6 MV photon beams on a Varian linear accelerator with sliding window technique. The technical solution of choice was a 5 field arrangements ('class solution') for most patients (n = 100); 6 fields were applied in 7, 7 fields in 8 patients.

Patient alignment was checked before radiation by portal imaging. Deviations of > 2 mm in nasopharyngeal cancers and paranasal sinus tumors, of > 3 mm in all other tumors, respectively, were corrected before treatment. Three-dimensional position deviations from the digitally reconstructed radiographs (DRRs) were compared and calculated automatically (lateral and axial deviation, rotation).

In the first 30 patients treated with IMRT at our institution, the accepted deviation was only 2 mm, independent of the diagnosis. The position in all patients used to be checked on a daily base for the entire treatment time and was prospectively analysed.

Deviations of >2 mm occurred in 108 out of 241 evaluated treatment sessions in patients (1:2.2 incorrect-to-correct position-ratio); 2/3 of all deviations that required a pre-treatment correction were observed in patients with large fields (when lymphatic pathways included in the treatment volume).

Based on those data we went over to a) an accepted 3 mm deviation for all patients except of those with sinonasal and nasopharyngeal tumors, and b) to the following portal vision check rhythm: daily checks only in the first three treatment days, followed by a once to twice a week portal vision check in all patients in whom positioning is initially found in the tolerated range. Every correction was followed by another daily check period of three days.

The dose homogeneity within the PTV was aimed to be in close accordance with the RTOG guidelines:

The dose was normalized to the mean dose in PTV1 which corresponds, in the majority of cases, approximately to the 95 % dose level in that volume.

- The prescription dose is the isodose which encompasses at least 95 % of the PTV

- no more than 20 % of any PTV will receive >110 % of it's prescribed dose

- no more than 1 % of PTV1 will receive < 93 % of its prescribed dose

- no more than 1 % or 1 cc of the tissue outside the PTV will receive > 110 % of the dose prescribed to the primary PTV

### Clinical quality assurance (QA)

#### - Follow up

During the course of irradiation, all patients were clinically assessed at regular weekly intervals, and 2 weeks and 2 months after completion of treatment.

Approximately 6 weeks after completion, all patients were also seen regularly in our joint clinics at the Department of Head and Neck Surgery or Maxillofacial Surgery. Further follow up visits were scheduled every 2 – 3 months in the first 2 years, 3 – 4 monthly in the third year. When clinical and/or endoscopic examination showed no evidence of disease no radiological investigations were performed; suspect findings were specified with CT-PET, suspect lymph nodes by needle aspiration and/or biopsy, respectively.

#### - QA with respect to posttreatment events

Isodose plans of all patients who experienced loco-regional failure or grade 3/4 late term effects were reviewed at the radiation planning work station, in order to check local dose distributions at the regions of interest.

#### - QA with respect to quality of life (QoL)

Toxicity was assessed based on SOMA LENT and RTOG/EORTC Radiation Morbidity Score. Both classifications have been considered; for simplification, grade 3 or 4 late reactions were termed 'grade 3/4' reactions.

Patients' QoL was prospectively assessed prior, during the course of radiation, and 2 weeks, then 2, 6, 12, 18, and 24 months following IMRT (EORTC/RTOG-QLQ; results in preparation).

StatView^® ^program Version 4.5 was used for calculation of Kaplan Meier actuarial survival curves. Mann-Whitney-U test was used for comparison of volumes. P values < 0.05 were considered statistically significant.

## Declaration of competing interests

The author(s) declare that they have no competing interests.

## Authors' contributions

GS and CG designed the study and analysed the data, GS carried out the data collection and drafted the manuscript. PH participated in collecting data and created the data base. BD reviewed and corrected the manuscript, BD and GK participated in drafting the 'methods'. UML reviewed and corrected the manuscript. All authors read and approved the final manuscript. The authors are the responsible physicians and physicists for the IMRT program.
